# Classification of dendritic cell phenotypes from gene expression data

**DOI:** 10.1186/1471-2172-12-50

**Published:** 2011-08-29

**Authors:** Giacomo Tuana, Viola Volpato, Paola Ricciardi-Castagnoli, Francesca Zolezzi, Fabio Stella, Maria Foti

**Affiliations:** 1 Genopolis Consortium, University of Milano-Bicocca, Milan, 20126, Italy; 2 Department of Informatics, Systems and Communication, University of Milano-Bicocca, Viale Sarca 336, 20126 Milano, Italy; 3 Singapore Immunology Network, Singapore, 138648, Singapore; 4 Department of Biotechnology and Bioscience, University of Milano-Bicocca, Milan, 20126, Italy

## Abstract

**Background:**

The selection of relevant genes for sample classification is a common task in many gene expression studies. Although a number of tools have been developed to identify optimal gene expression signatures, they often generate gene lists that are too long to be exploited clinically. Consequently, researchers in the field try to identify the smallest set of genes that provide good sample classification. We investigated the genome-wide expression of the inflammatory phenotype in dendritic cells. Dendritic cells are a complex group of cells that play a critical role in vertebrate immunity. Therefore, the prediction of the inflammatory phenotype in these cells may help with the selection of immune-modulating compounds.

**Results:**

A data mining protocol was applied to microarray data for murine cell lines treated with various inflammatory stimuli. The learning and validation data sets consisted of 155 and 49 samples, respectively. The data mining protocol reduced the number of probe sets from 5,802 to 10, then from 10 to 6 and finally from 6 to 3. The performances of a set of supervised classification models were compared. The best accuracy, when using the six following genes --Il12b, Cd40, Socs3, Irgm1, Plin2 and Lgals3bp-- was obtained by Tree Augmented Naïve Bayes and Nearest Neighbour (91.8%). Using the smallest set of three genes --Il12b, Cd40 and Socs3-- the performance remained satisfactory and the best accuracy was with Support Vector Machine (95.9%). These data mining models, using data for the genes Il12b, Cd40 and Socs3, were validated with a human data set consisting of 27 samples. Support Vector Machines (71.4%) and Nearest Neighbour (92.6%) gave the worst performances, but the remaining models correctly classified all the 27 samples.

**Conclusions:**

The genes selected by the data mining protocol proposed were shown to be informative for discriminating between inflammatory and steady-state phenotypes in dendritic cells. The robustness of the data mining protocol was confirmed by the accuracy for a human data set, when using only the following three genes: Il12b, Cd40 and Socs3. In summary, we analysed the longitudinal pattern of expression in dendritic cells stimulated with activating agents with the aim of identifying signatures that would predict or explain the dentritic cell response to an inflammatory agent.

## Background

Genome-wide screening of expression profiles has provided a broad perspective on gene regulation in health and disease. Gene expression is controlled over a wide range through complex interplay between DNA regulatory proteins, microRNA molecules and epigenetic modifications determining transcript production [1-3]. For example, gene expression profiles in mouse dendritic cells (DCs) in response to microbial organisms and their components have been studied using a functional genomics approach and the molecular patterns involved in DCs activation have been determined [4-7]. However, the high-dimensionality inherent in genome-wide analyses makes it difficult to extract biologically useful information from gene expression data. Early attempts at genome-wide expression analysis used unsupervised methods to identify groups of genes or conditions with similar expression profiles [8-10]; the observation that functionally related or co-regulated genes often cluster together was used to provide biological insight. Classification studies in the field of microarray analysis have become important for the development of diagnostic tests. One of the most common approaches for supervised classification is binary classification, which distinguishes between two types of phenotype: positive, for example compound A-treated samples, and negative, often control or compound B-treated samples. A collection of samples with known type labels is used to train a classifier that is then used to classify new samples. For example, the supervised classification models Support Vector Machines [11], Classification Trees [12] and Artificial Neural Networks [13] have led to the generation of functional gene signatures for haematological malignancies [8,14-16], and for the identification of molecular markers that provide accurate diagnosis, prognosis and selection of treatment regimens for human diseases [17-20]. These methods are able to identify genes and, consequently gene networks, associated with particular phenotypes. More recently, supervised classification models combining cross validation and heuristic search strategies have been used to discover optimal expression signatures in cancer [21-23]. However, despite the number of classification methods that have been developed for this kind of knowledge extraction, such knowledge has not yet been widely used in diagnostic or prognostic decision-support systems [13]. This is partly due to the variability of the results obtained [24] and also to the different data sets used [25,26].

Few methods have been used to identify specific expression signatures that could contribute to the molecular diagnosis of inflammatory-based diseases. The Random Forests method has been used to generate a 44-gene signature in DCs to distinguish between inflammatory and non-inflammatory stimuli, but this gene signature is too large for clinical exploitation [5]. Here, we report a data mining protocol developed through the analysis of a database generated from microarray experiments with DCs exposed to various stimuli able to induce cell activation. This protocol allowed the selection of a small set of genes which were subsequently used by supervised classification models to make inferences concerning the inflammatory state of the samples.

## Results

**Figure 1:**
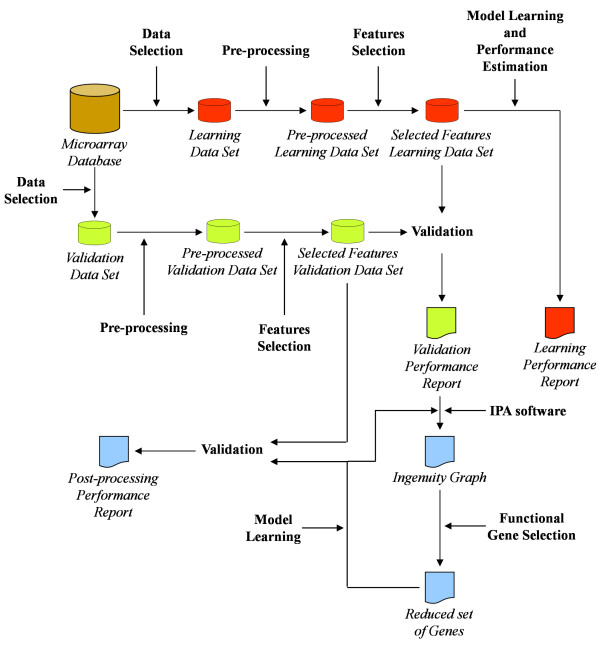
**Knowledge Extraction Protocol**. Data are selected (Data Selection) from the Microarray Database to obtain the Learning Data Set which pre-identifies the relevant genes (Selected Features Learning Data Set). Selected genes are used to train several DM models whose performance (Model Learning and Performance Estimation) is summarised (Learning Performance Report). DM models are validated (Validation) to obtain the Validation Performance Report, and the selected genes are used to query the Ingenuity Pathway Analysis software (IPA software). Functional Gene Selection exploits the Ingenuity Graph to obtain the Reduced set of Genes that is used for Model Learning. The Validation task generates the Post-processing Performance Report.

The Knowledge Extraction Protocol (KEP), depicted in Figure 1, was used to select relevant probe sets (genes) and to train supervised classification models to discriminate between "inflammatory" and "not inflammatory" phenotypes of DCs.

### Data Selection

*Mouse data*: two microarray data sets, namely the *Learning Data Set *and the *Validation Data Set*, were defined. The *Learning Data Set *included the results obtained from microarray experiments performed with: Affymetrix MGU74Av2 arrays (89 samples - 9 different stimuli) [5], Affymetrix MOE430A arrays (44 samples - 4 different stimuli) and MOE430A 2.0 arrays (22 samples - 2 different stimuli). The *Validation Data Set *the results of microarray experiments performed with: Affymetrix MGU74Av2 arrays (43 samples - 6 different stimuli) [5] and MOE430A 2.0 arrays (6 samples - 1 stimulus; this stimulus is the only one that was not with the DC cell line D1 [27], but used bone marrow-derived DCs (BMDC) [28]).

### Pre-processing

The differences in array formats required the data to be standardised. GeneChip Mouse Expression 430 (MOE430A 2.0) is the latest version of Affymetrix mouse arrays and contains 22,600 probe sets. All the probe sets of the MOE430A array are included in the MOE430A 2.0 array. The older mouse array, MGU74Av2, contains 12,488 probe sets that only partially match the probe sets of its more recent releases. Affymetrix provides "best match" probe set tables which allow the mapping of equivalent probe sets between different array releases.

The following pre-processing steps were performed: **a) **Probe set best matching between MOE430A and MGU74Av2. This resulted in 8,904 probe sets, also included in the MOE430A 2.0 array; **b) **Probe set filtering based on Affymetrix *grading A *annotation. This step retained 8,349 probe sets out of the 8,904 available; **c) **Probe set filtering based on expression signals. Every probe set whose expression signal was below 100 was discarded, such that 5,802 probe sets of the 8,349 available were retained; **d) **per sample Z-score computation.

The pre-processing procedure generated the *Pre-processed Learning Data Set*, which consisted of 155 samples (15 different stimuli), and the *Pre-processed Validation Data Set*, which consisted of 49 samples (7 different stimuli). Both data sets contained the same 5,802 probe sets. The class counts for the two data sets are summarised in Table 1 and the detailed list of the experiments and array types is reported in Additional file 1.

**Table 1:** Frequency of the class variable for Pre-processed Data Sets.

Pre-processed Data Set	Inflammatory	Not Inflammatory
Learning	106	49
Validation	32	17

### Feature Selection

Feature selection involves the identification and removal of *non significant features*. The probe sets which provide no information helping to discriminate between "inflammatory" and "not inflammatory" states of the samples are thereby removed from the analysis.

The Weka software environment was used for feature selection [29]. The feature selection task was performed through an ADTree-based wrapper schema (default parameter values) applied to the *Pre-processed Learning Data Set*. This step selected an expression signature of ten probe sets (Table 2) from among the initial 5,802, which generated the *Selected Features Learning Data Set*.

**Table 2:** Selected Genes.

Feature Name	Chromosome	Gene Symbol	Gene Title	Entrez Gene ID
101481_at1415791_at	5	Rnf34	ring finger protein 34	80751
92232_at-1416576_at	11	Socs3	suppressor of cytokine signaling 3	12702
97409_at-1418825_at	11	Irgm1	immunity-related GTPase family M member 1	15944
100779_at-1419530_at	11	Il12b	interleukin 12b	16160
93347_at-1421873_s_at	13	Rab24	RAB24, member RAS oncogene family	19336
102062_at-1423416_at	9	Smarcc1	SWI/SNF related, matrix associated, actin dependent regulator of chromatin, subfamily c, member 1	20588
103260_at-1430291_at	14	Dock5	dedicator of cytokinesis 5	68813
98589_at-1448318_at	4	Plin2	perilipin 2	11520
97507_at-1448380_at	11	Lgals3bp	lectin, galactoside-binding, soluble, 3 binding protein	19039
92962_at-1449473_s_at	2	Cd40	CD40 antigen	21939

### Model Training and Performance Estimation

This task, implemented through the Weka software environment, used the *Selected Features Learning Data Set *to train, evaluate and compare the performance of the following supervised classification models: *ZeroR*, *IB*-3, *C4.5*, *Logistic*, *Multi Layer Perceptron *(*MLP*), *Naïve Bayes *(*NB*), *Random Forest *(*RF*), *Support Vector Machines *(*SMO*-puk) and *Tree Augmented Naïve bayes *(*TAN*).

These models were chosen because they are state-of-the-art for solving supervised classification problems. *ZeroR *uses the majority criteria to classify a sample, i.e. it classifies each sample according to the majority of the class distribution. The weighted averages, estimated through ten repeated 10-fold cross validations, of the following performance measures are reported in Table 3: *Precision*, *Recall*, *F-measure*, *ROC *and *Accuracy*. *ZeroR *was used as the baseline measure of performance, and the performance of the other models was assessed from ROC values: the ROC values were 97.5% for each *C4.5*, 100% for *MLP*99.9% for *IB*-3 99.8% for *RF*, 99.0% for *SMO*-puk, and 99.2% for *TAN*, and 98.6% for both *Logistic *and *NB*. However, using accuracy to compare the supervised classification models, a different picture is obtained. The model with the highest accuracy value was *RF *(99.1%). The other accuracy values were 98.6% for both *SMO*-puk and *MLP*, 98.1% for *IB*-3, 96.3% for both *TAN *and *C4.5*, 95.5% for *Logistic *and 94.2%, the lowest value, for *NB*.

**Table 3:** Learning Performance Report.

	Precision			Recall			F-measure			ROC			Accuracy		
	min	**mid**	max	min	**mid**	max	min	**mid**	max	min	**mid**	max	min	**mid**	max
ZeroR	46.8	**46.8**	46.8	68.4	**68.4**	68.4	55.5	**55.5**	55.5	47.9	**47.9**	47.9	68.4	**68.4**	68.4
IB-3	97.4	**98.1**	98.7	97.4	**98.1**	98.7	97.4	**98.1**	98.7	99.9	**99.9**	100.0	97.4	**98.1**	98.7
C4.5	94.2	**96.3**	98.1	94.2	**96.3**	98.1	94.1	**96.3**	98.1	92.9	**97.5**	98.9	94.2	**96.3**	98.1
Logistic	94.4	**95.6**	96.9	94.2	**95.5**	96.8	94.2	**95.5**	96.8	98.3	**98.6**	98.9	94.2	**95.5**	96.8
MLP	98.2	**98.7**	98.8	98.1	**98.6**	98.7	98.1	**98.6**	98.7	99.9	**100.0**	100.0	98.1	**98.6**	98.7
NB	93.7	**94.4**	95.1	93.5	**94.2**	94.8	93.6	**94.2**	94.9	98.3	**98.6**	98.9	93.6	**94.2**	94.8
RF	96.8	**99.1**	100.0	96.8	**99.1**	100.0	96.7	**99.1**	100.0	98.5	**99.8**	100.0	96.8	**99.1**	100.0
SMO-puk	98.2	**98.7**	98.8	98.1	**98.6**	98.7	98.1	**98.6**	98.7	98.6	**99.0**	99.1	98.1	**98.6**	98.7
TAN	94.9	**96.3**	98.1	94.8	**96.3**	98.1	94.8	**96.2**	98.1	98.9	**99.2**	99.4	94.8	**96.3**	98.1

### Validation

Supervised classification models, which generate the selected gene expression signature, need to be able to classify data sets other than the one they were trained on if they are to be useful. Therefore, the performance of the supervised classification models was evaluated by exploiting the *Selected Features Validation Data Set *(Table 4). The Bayesian models, *NB *(93.0%) and *TAN *(92.8%), attained the highest ROC values and both *IB*-3 (92.6%) and *C4.5 *(91.2%) gave good ROC values. However, the ROC values were substantially lower for *RF *(89.6%), *MLP *(88.1%), *SMO*-puk (86.7%) and *Logistic *(86.6%). The *ZeroR *model gave an ROC value of 50% confirming, as was expected, that it behaves like a random guessing model. A different picture emerged when the accuracy performance measure was used. Indeed, the best accuracy value (93.9%) was for *C4.5 *and *RF*. The accuracy value for the *TAN *model was 91.8% and that for *SMO*-puk was 89.8%. The accuracy values were lower for *NB *(87.8%), *IB*-3 (85.7%) and *Logistic *(81.6%). The model with the worst accuracy value was *MLP *(77.6%).

**Table 4:** Validation Performance Report.

	Precision	Recall	F-measure	ROC	Accuracy	Errors
ZeroR	42.6	65.3	51.6	50.0	65.3	17/49
IB-3	85.6	85.7	85.6	92.6	85.7	07/49
C4.5	94.4	93.9	93.7	91.2	93.9	03/49
Logistic	81.9	81.6	80.7	86.6	81.6	09/49
MLP	77.2	77.6	76.4	88.1	77.6	11/49
NB	87.7	87.8	87.6	93.0	87.8	06/49
RF	93.9	93.9	93.8	89.6	93.9	03/49
SMO-puk	90.1	89.8	89.5	86.7	89.8	05/49
TAN	91.9	91.8	91.7	92.8	91.8	04/49

### Functional Gene Selection

**Figure 2:**
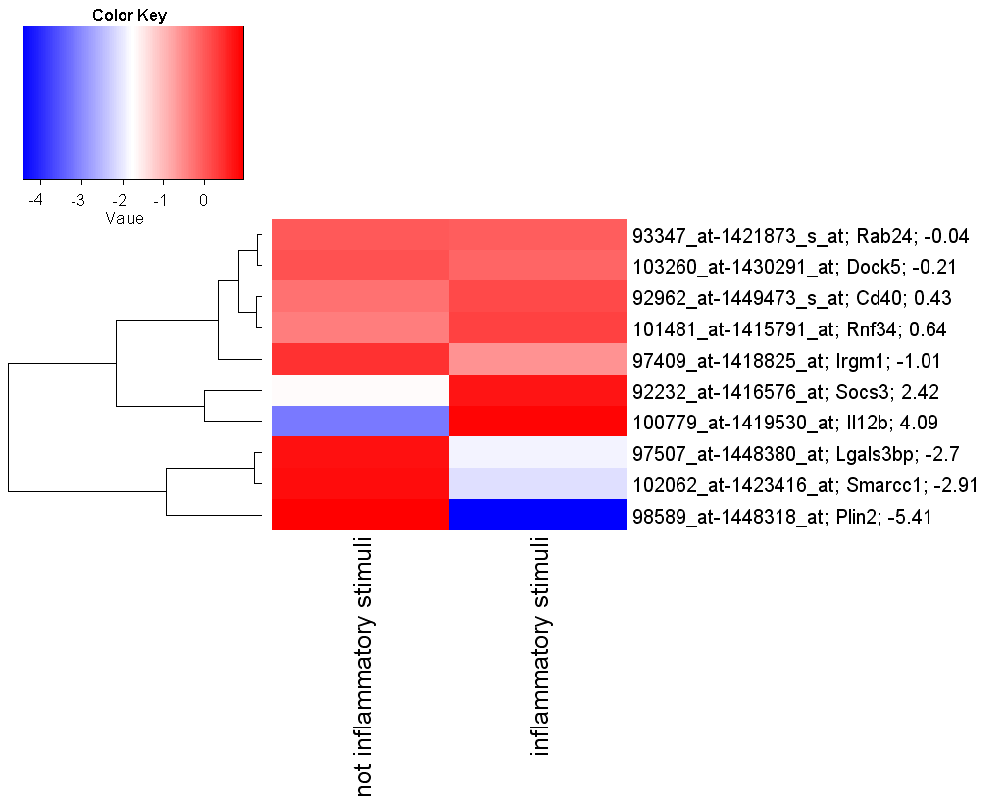
**Heatmap of the 10 selected genes calculated on the testing set**. The heatmap reports the ratio between the log2 mean expression value for each condition and the median values of each probe set. The LogFC value is reported next to the Gene Symbol for each gene. Red indicates up-regulated and blue down-regulated probe sets. The probe sets (rows) are grouped according to their similarity by hierarchical clustering using complete linkage (Euclidean distance).

**Figure 3:**
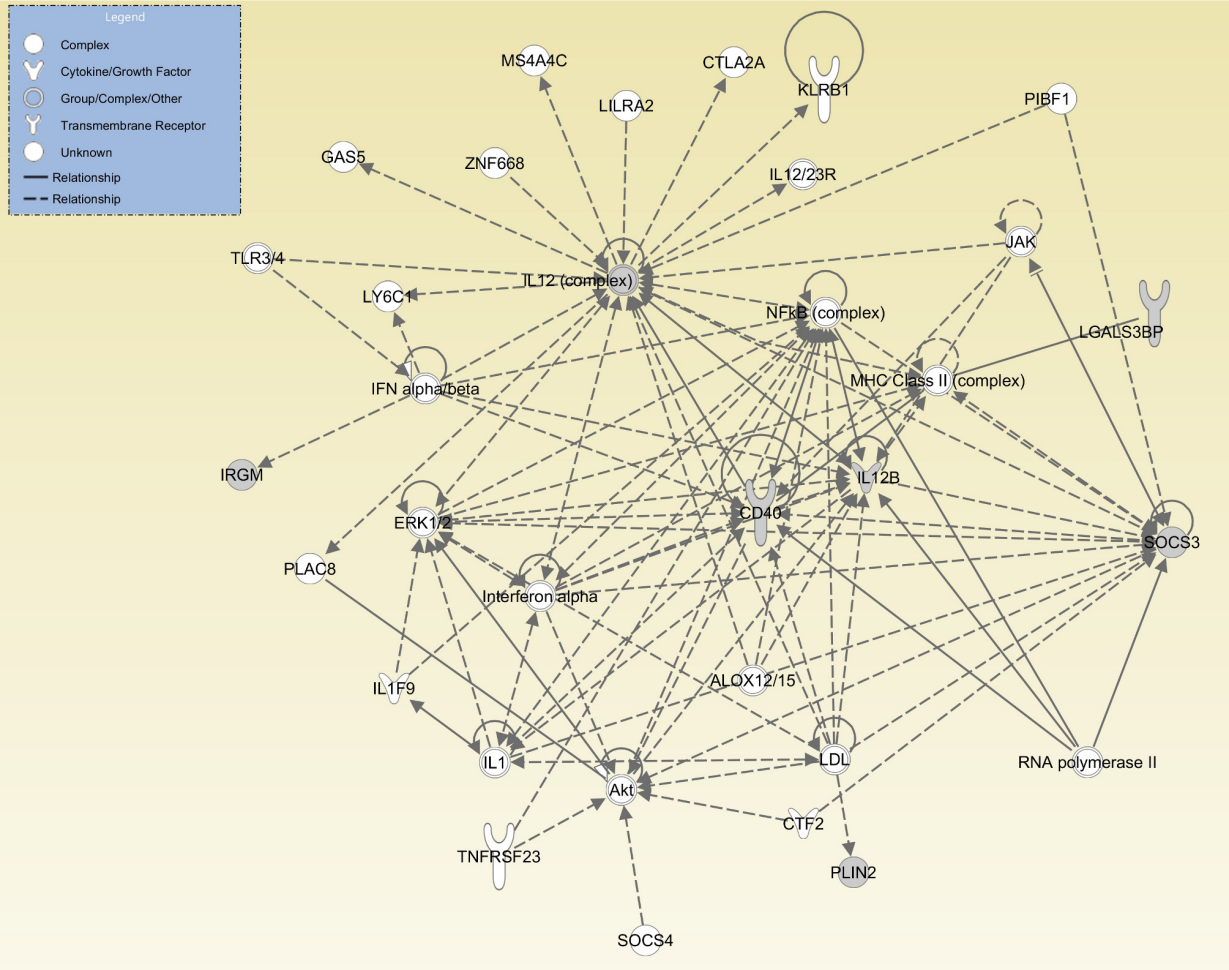
**Top IPA generated network**. The figure illustrates the graphical representation of the Ingenuity Pathway Analysis software. Each node contains comprehensive information on a gene's function, how that gene is regulated, its direct neighbours, and synonyms, Genes are represented as nodes and the biological relationship between two nodes is represented as an edge: dashed lines if relationship is indirect and continuous lines are for direct relationships. Nodes are displayed using various shapes that represent the functional class of the gene product (legend in the top left). The output of the IPA query is exploited for Biological Annotation from the IPA Knowledge Database. The top network found by IPA concerns cellular growth and proliferation and the humoral immune response. Six (grey nodes) among the ten input genes show more than one interaction (also indirect) in the network built by IPA.

The annotations of the ten selected genes (Table 2) indicate that four, namely Socs3, Irgm1, Il12b and Cd40, are associated with known immune-related functions. Expression of six of the ten selected genes differs between the "non inflammatory" and "inflammatory" classes with an absolute Log2 FoldChange (LogFC) greater than 1. A heatmap (Figure 2) was established for the LogFC of the average signal intensities of the selected genes for the "non inflammatory" and "inflammatory" experiments, calculated on the median expression value for that gene. Il2b and Socs3 are up-regulated with LogFC values of 4.1 and 2.7, respectively. Irgm1, Plin2, Lgals3bp and Smarcc1 are down-regulated with LogFC values of -1.1, -5.6, -2.7 and -2.9, respectively in the samples induced with inflammatory stimuli. The remaining four genes, namely Cd40, Dock5, Rnf34 and Rab24, show a level of up-regulation or down-regulation resulting in a value of LogFC which is smaller than 1. To characterize the selected gene expression signature further, the ten genes were examined with Ingenuity^® ^Pathway Analysis (IPA) software and the Ingenuity^® ^Knowledge Base (IKB). The IPA software was queried to find the biological interactions (direct and indirect) among the ten genes. The top network retrieved (IPA score equal to 16), depicted in Figure 3, contains six genes of the selected gene expression signature (grey nodes in Figure 3) and 25 further genes (white nodes in Figure 3) that were added by the IKB to build the network. The biological functions associated with this network are the following: *Cellular Growth and Proliferation*, *Haematological System Development and Function*, *Humoral Immune Response*.

The molecular and cellular functions of the genes included in the selected gene expression signature were analysed with IPA (Table 5). This identified the *Infection Mechanism *to be the top function related to "Diseases and Disorders", the *Cellular Growth and Proliferation *to be the top function related to "Molecular and Cellular Functions" and the *Haematological System Development and Function *to be the top function related to "Physiological System Development and Function".

**Table 5:** Biological functions related to the selected genes.

*Diseases and Disorders*		
Name	p-value	# Molecules
Infection Mechanism	4.32E-07 - 4.79E-02	4
Genetic Disorder	1.30E-06 - 4.33E-02	4
Hematological Disease	1.30E-06 - 1.91E-02	5
Immunological Disease	1.30E-06 - 4.27E-02	5
Gastrointestinal Disease	1.33E-06 - 3.80E-02	4

***Molecular and Cellular Functions***		

**Name**	**p-value**	**# Molecules**

Cellular Growth and Proliferation	1.23E-07 - 4.72E-02	5
Lipid Metabolism	4.32E-06 - 2.05E-02	4
Small Molecule Biochemistry	4.32E-06 - 4.13E-02	5
Cell Signaling	4.35E-06 - 4.13E-02	4
Cellular Development	6.63E-06 - 4.86E-02	5

***Physiological System Development and Function***		

**Name**	**p-value**	**# Molecules**

Hematological System Development and Function	1.23E-07 - 4.86E-02	6
Tissue Development	1.23E-07 - 3.93E-02	6
Humoral Immune Response	4.32E-07 - 2.99E-02	2
Organismal Survival	6.36E-06 - 4.67E-04	5
Cell-mediated Immune Response	6.63E-06 - 3.86E-02	5

**Figure 4:**
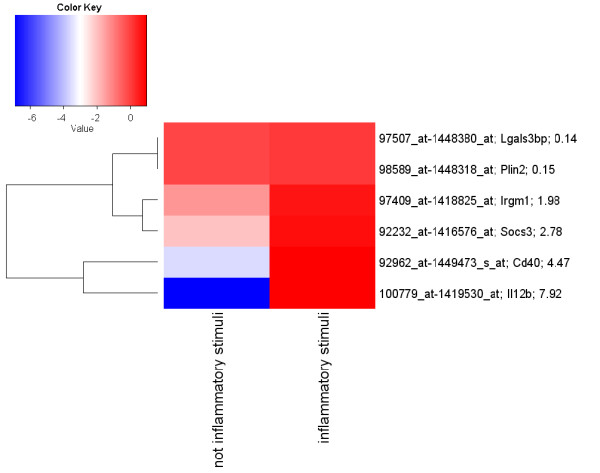
**Heatmap of the six selected genes calculated on the validation data set**. The heatmap reports the ratio between the log2 mean expression value for each condition and the median values of each probe set. The LogFC values are reported next to the Gene Symbol for each gene. Red indicates up-regulated and blue down-regulated probe sets. The probe sets (rows) are grouped according to their similarity by hierarchical clustering using complete linkage (Euclidean distance).

A smaller set of genes (Table 6) was obtained by removing those genes not included in the IPA top network (Figure 3). The performances of the classification models which exploit this reduced set of genes on the *Selected Features Validation Data Set *are reported in Table 7. The ROC values of *RF*, *MLP*, *SMO*-puk and *IB*-3 were not significantly affected by the functional gene selection step. However, the ROC values for *NB*, *TAN *and *C4.5 *increased whereas that for *Logistic *decreased. The accuracy values of *TAN*, *SMO*-puk and *NB *were not affected by the functional gene selection step; they increased from 85.7% to 91.8% for *IB*-3, from 77.6% to 81.6% for *MLP *and from 81.6% to 83.7% for *Logistic*, but decreased from 93.9% to 85.7% for both *C4.5 *and *RF*. The heatmap in Figure 4 shows the modulation of the six genes in the *Selected Features Validation Data Set*. Il2b, Socs3 and Cd40 were up-regulated in the *Selected Features Validation Data Set *also; with Cd40 being up-regulated (LogFC = 4.5) in the *Selected Features Validation Data Set *in comparison with the *Selected Features Learning Data Set *(LogFC = 0.45). Furthermore, Irgm1 was up-regulated (LogFC = 1.9) in the *Selected Features Validation Data Set *but down-regulated in the *Selected Features Learning Data Set *(LogFC = -5.6). Plin2, Lgals3bp and Smarcc1 were not modulated in the *Selected Features Validation Data Set *but were down-regulated in the *Selected Features Validation Data Set *(Figure 2). The best classification models, i.e. *IB-3 *and *TAN*, misclassified four of the 49 samples belonging to the *Selected Features Validation Data Set*. One sample was genuinely allocated to the wrong group, whereas two were known to be labelled with the wrong class and one was known to be an outlier.

**Table 6:** Reduced set of Genes.

Feature Name	Chromosome	Gene Symbol	Gene Title	Entrez Gene ID
92232_at-1416576_at	11	Socs3	suppressor of cytokine signaling 3	12702
97409_at-1418825_at	11	Irgm1	immunity-related GTPase family M member 1	15944
100779_at-1419530_at	11	Il12b	interleukin 12 b	16160
98589_at-1448318_at	4	Plin2	perilipin 2	11520
97507_at-1448380_at	11	Lgals3bp	lectin, galactoside-binding, soluble, 3 binding protein	19039
92962_at-1449473_s_at	2	Cd40	CD40 antigen	21939

**Table 7:** Post-processing Performance (Functional Gene Selection I).

	Precision	Recall	F-measure	ROC	Accuracy	Errors
ZeroR	42.6	65.3	51.6	50.0	65.3	17/49
IB-3	91.8	91.8	91.8	90.8	91.8	04/49
C4.5	86.8	85.7	85.9	86.3	85.7	07/49
Logistic	84.3	83.7	83.9	91.5	83.7	08/49
MLP	82.8	81.6	81.9	86.8	81.6	09/49
NB	87.8	87.8	87.8	89.9	87.8	06/49
RF	86.8	85.7	85.9	90.1	85.7	07/49
SMO-puk	89.7	89.8	89.7	88.1	89.8	05/49
TAN	91.9	91.8	91.7	90.1	91.8	04/49

**Figure 5:**
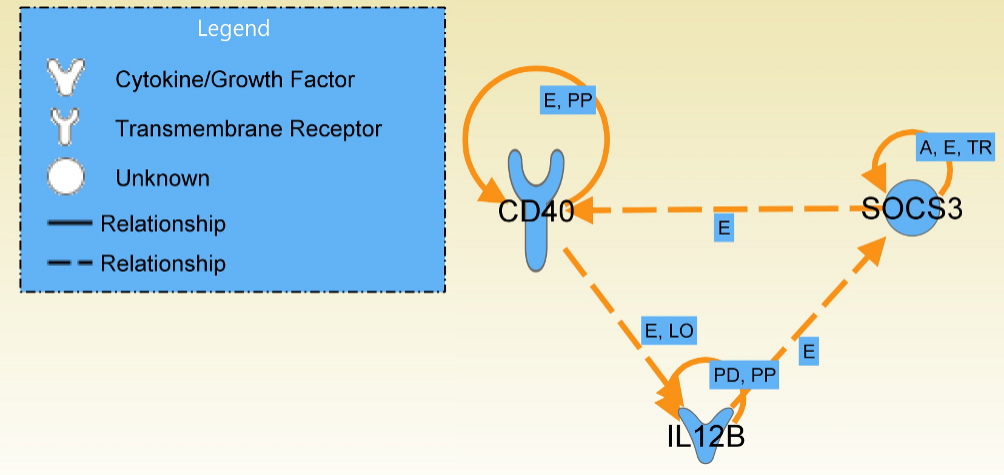
**IPA functional relationship among the 3 selected features**. Genes are represented as nodes and the biological relationships between two nodes are represented as edges: dashed lines indicate that the relationship is indirect. (A) Activation, (E) Expression (includes metabolism/synthesis for chemicals), (LO) Localisation, (PD) Protein-DNA binding (PP) Protein-Protein binding, (TR) Translocation.

Reducing the number of genes from ten to six on the basis of the information derived from the top network generated by IPA gave satisfactory accuracy values. Therefore, a further Functional Gene Selection step was performed. Three of the selected genes were directly linked to each other in the IPA top network: Cd40, Il12b and Socs3 (Figure 5). The results of the Validation task, when only the above genes were used, are reported in Table 8. The model that giving the best accuracy value was *SMO*-puk (95.9%). The second best accuracy value (91.8%) was with *IB*-3 and *NB*. *Logistic *and *TAN *gave the same, satisfactory, accuracy value (89.8%). That for *MLP *was 87.8% and the lowest value (85.7%) was for *C4.5 *and *RF*. The best model, i.e. *SMO*-puk, misclassified two of the 49 samples. These samples were those known to be labelled in the wrong class. These findings confirm that the three genes are sufficient for correct classification of all the samples of the *Selected Features Validation Data Set*.

**Table 8:** Post-processing Performance (Functional Gene Selection II).

	Precision	Recall	F-measure	ROC	Accuracy	Errors
ZeroR	42.6	65.3	51.6	50.0	65.3	17/49
IB-3	91.9	91.8	91.7	93.5	91.8	04/49
C4.5	85.6	85.7	85.6	83.5	85.7	07/49
Logistic	89.7	89.8	89.7	91.5	89.8	05/49
MLP	88.3	87.8	87.9	92.5	87.8	06/49
NB	91.8	91.8	91.8	93.2	91.8	04/49
RF	85.6	85.7	85.6	84.7	85.7	07/49
SMO-puk	96.2	95.9	95.9	94.1	95.9	02/49
TAN	90.0	89.8	89.9	89.3	89.8	05/49

### A 3-gene signature associated with inflammation in Human Dendritic Cells

Human Data. To test the general applicability of the proposed protocol, Affymetrix HGU133A gene expression microarray data for 27 human samples (corresponding to nine time series) was used to validate the performance of the 3-gene signature classifiers, also in human dendritic cells. A data set for human monocyte-derived dendritic cells treated with *Mycobacteria tuberculosis *was derived from a previous study [30] and tested (Table 9). All the supervised classification models, with the exception of IB-3 and SMO-puk, achieved an accuracy of 100% indicating that the 3-gene signature selected on mouse DCs indeed corresponds to a general signature of inflammation in dendritic cells in both human and mouse systems. Therefore, we suggest CD40, Il12b and Socs3 can be considered to be the master genes of inflammation and activation in DCs.

**Table 9:** Performance of 3-genes signature classifiers on the human data set.

	Accuracy	Errors
IB-3	92.6	2/27
C4.5	100.0	0/27
Logistic	100.0	0/27
MLP	100.0	0/27
NB	100.0	0/27
RF	100.0	0/27
SMO-puk	71.4	8/27
TAN	100.0	0/27

## Discussion

In this study, we used advanced supervised analysis to derive specific transcriptional signatures from differentially activated DCs and assessed whether this molecular signatures can define DCs phenotypes in vitro. DCs form the connection between innate and adaptive mechanisms of the immune system. Studies in mice have demonstrated that cellular vaccination with antigen-bearing DCs is efficient in stimulating antigen-specific T cell responses. Because of the immune-regulating functions of DCs, the therapeutic use of DCs in medicine to control immune responses is an attractive strategy. DCs are indeed regarded as a powerful tool for anti-cancer immunotherapy [31]. In addition, to treat patients suffering from autoimmune or inflammatory diseases, it is desirable to downregulate immune responses in an antigen-specific or a tissue-specific manner without causing systemic immunosuppression. Moreover, graft-versus-host disease (GVHD) and graft rejection are the most serious problems in transplantation medicine, and control of alloreactive immune responses is the key to overcoming these problems. Therefore, antigen-specific negative regulation by DCs with immunosuppressive function is considered to be a promising treatment method also in the field of transplantation medicine [32,33]. In summary, a number of studies describe the generation of DCs from sources aiming at cell therapy [34,35]. Nevertheless, no methods exist today to test quality of the cell type generated. Therefore, a molecular test that could confirm DCs quality before their use in clinic will provide valuable information into the field of DCs therapies.

The problem of sample classification via gene signatures derived from transcriptional profiling has received increasing attention in the context of DNA microarrays. We used various aspects of the evaluation of gene selection approaches by combining the analysis of different markers of performance. First, we selected a list of genes, from whole-genome profiling of DCs, able to discriminate DC activation state. Second, to reduce the bias due to the classification model, we estimated different parameters through optimisation on an independent validation data set.

The Knowledge Extraction Protocol (KEP) (Figure 1) selected ten genes that, on the *Selected Features Validation Data Set*, discriminated between "inflammatory" and "not inflammatory" stimuli with an accuracy of 93.9% for *C4.5 *and *RF *and of 91.8% for *TAN*.

Six of the ten genes selected were modulated in the *Selected Features Learning Data Set *between the "not inflammatory" and "inflammatory" classes with an absolute Log2FoldChange (LogFC) greater than 1. The heatmap of the selected genes is shown in Figure 2 and revealed that two of them were up-regulated and four were down-regulated. Il2b, Socs3 and Cd40 were up-regulated (Figure 4) also in the *Selected Features Validation Data Set*; notably, Cd40 was up-regulated (4.5 LogFC) in the inflammatory state samples of the *Selected Features Validation Data Set*, compared to 0.45 LogFC in the *Selected Features Learning Data Set*. Plin2, Lgals3bp and Smarc1 were not substantially modulated in the *Selected Features Validation Data Set *and were down-regulated in the *Selected Features Learning Data Set*. Modulation of these selected genes should be further investigated biologically to validate these findings.

KEP misclassified four of the 49 samples of the *Selected Features Validation Data Set*; one sample was derived from D1 cells treated with the *Listeria monocytogenes *EGD for 4 h replicate A, and three samples from D1 treated with the *Listeria innocua *0 h replicates A and B and 8 h replicate A. The two time 0 h samples of the *Listeria innocua *experiment were known to be mislabelled, and the sample 8 h was found to be an outlier. Hierarchical clustering analysis of the samples from this *Listeria monocytogenes *EGD experiment did not show any anomaly that might provide an explanation for the misclassification (data not shown). Remarkably, in the *Selected Features Validation Data Set*, samples from experiments involving cells from different sources (e.g. bone-marrow derived DCs) were not misclassified. This suggested that the KEP presented in this work may discriminate inflammatory signatures for DCs from diverse sources.

Several methods, including traditional statistical techniques and state of the art computer-intensive methodologies, have been investigated to predict inflammatory signatures in DCs. Activation of DCs with LPS and with IFN-β have been shown to generate cells prone to produce Th1 attractants that are effective for adoptive immune cancer therapy [36,37]. It has been also demonstrated that DCs exposed to supernatants derived from tumours treated with some cytotoxic drugs are capable to modulate co-stimulatory markers and to trigger T cell responses [38]. A 44-gene signature in DCs, able to discriminate between different functional states, is described in [5]. Here, we report a significant improvement over the previous work by reducing the number of genes in the signature and by testing their performance with DCs derived from different hosts, namely mouse and human. We selected a signature of inflammation based on the expression of ten genes and demonstrated that this list could be further reduced to three genes without significantly affecting the classification performance. The three genes, namely CD40, Il12b and Socs3, can thus be considered to be the master genes of activation/inflammation in DCs. CD40 mediates a broad variety of immune and inflammatory responses, and the ligand-receptor interaction is responsible for immune activation; Il12b is a part of the IL12 cytokine complex, a cytokine that acts on T and natural killer cells, and has a broad range of biological activities, the most important being the induction of Th1 cells development; the Socs3 gene encodes a member of the STAT-induced STAT inhibitor (SSI) family, also known as the suppressor of cytokine signalling (SOCS) family. SSI family members are cytokine-inducible negative regulators of cytokine signalling [39-42]. Therefore, the regulation of these genes in concert in DCs suggests that they may serve as molecular markers of inflammation/activation both in human and murine DCs.

## Conclusions

Experimental and bioinformatics strategies of this type may be used to improve treatment decisions for other inflammatory contexts, particularly chronic diseases. The whole-genome approach holds the promise to define the DCs functional quality that results in a better prediction of the stimulatory capacity of the cells. This approach may become a powerful strategy in personalised medicine.

## Methods

The Knowledge Extraction Protocol (Figure 1) is based on Data Mining (DM) [43,44] and consists of the following tasks; Data Selection, Pre-processing, Feature Selection, Model Training and Performance Estimation, Validation and Functional Gene Selection.

### Data Selection

*Mouse data*: all time-series experiments of the *Learning Data Set *used the murine cell line D1 [27] treated for 0, 2, 4, 8, 12 and 24 hours with "inflammatory" (CpG, *Shistosomula eggs*, LPS, *Leishmania promastigote*, Zymosan, polyIC, *Listeria monocytogenes*, *Listeria innocua*, *Bordetella pertussis*, *Bordetella parapertussis*, *Lactobacillus paracasei*, *Lactobacillus lactis*) and "not inflammatory" stimuli (*Shistosomula SLA*, *Leishmania amastigote*, dexamethasone) [5]. The *Validation Data Set *includes experiments performed with "not inflammatory" (cholera toxin) and "inflammatory" stimuli (*Listeria monocytogenes EGD-e, EGD-d, EGD-p, Listeria innocua*, LPS). Time 0 hours experiments were labelled as "not inflammatory". All the experiments were performed with D1 cells, with the exception of the LPS time series that was produced with bone marrow-derived murine DCs [27]. Most experiments were done on biological duplicates. Total RNA was extracted, labelled and hybridized to an Affymetrix GeneChip^® ^as described in [5].

*Human Data*: the human dataset used for the validation for human DCs was obtained from a previous study [30]. Briefly, human DCs were differentiated from human circulating monocytes and treated with *M. tuberculosis *H37Rv at multiplicity of infection of 1 for 4, 18 and 48 h. Total RNA was extracted, labelled and hybridised to a Human U133A Affymetrix GeneChip^® ^as described in [30].

For all the arrays, both with human and mouse sets, signal summarisation was performed using the *Affymetrix GeneChip Operating Software*^® ^(GCOS) and the MicroArray Suite version 5 (MAS 5.0) algorithm with scaling intensity target set to 100.

### Pre-processing

*Mouse Data*: three kinds of arrays (Affymetrix^® ^MOE430 2.0, MOE430A 2.0 and MGU74Av2) were used. All probe sets represented on the GeneChip^® ^MOE430A (22,690 probe sets) are included on the GeneChip^® ^MOE430A 2.0 array; the MG-U74Av2 array contains different probe sets (12,488 probe sets). The probe sets associated with the MOE430A, MOE430A 2.0 and MG-U74Av2 arrays mapped with the "mgu74v2_vs_mouse430_best_match" annotation table from Affymetrix http://www.affymetrix.com/support/technical/comparison_spreadsheets.affx?pnl = 1_2#1_2. Only the probe sets associated with the Affymetrix *annotation Grade *"A" were retained (8,349 probe sets). The pre-processing task removes from the *Learning Data Set/Validation Data Set *those probe sets associated with high levels of noise, and labels samples as *inflammatory *or *not inflammatory *and thus generates the *Pre-processed Learning Data Set/Pre-processed Validation Data Set*. The noisy probe sets are removed by using the probe set filter procedure which selects a probe set in the case where its signal exceeds 100 for at least two samples. The pre-processed data sets consisted of 5,802 features (probe sets). Note that the pre-processing task transforms the *Learning Data Set/Validation Data Set *in such a way that each measurement of a probe set, associated with a given point in time, becomes an observation in the corresponding *Pre-processed Learning Data Set/Pre-processed Validation Data Set*. The *Pre-processed Learning Data Set *consisted of 155 cases (15 stimuli, 30 time series) and the *Pre-processed Validation Data Set *consisted of 49 cases (7 stimuli, 12 time series). The counts of the class variables are reported in Table 1. Intensity data was used to compute per-sample Z-score.

*Human Data*: Affymetrix NetAffx tool http://www.affymetrix.com/index.affx was used to retrieve all human corresponding orthologous probe sets for Cd40, Il12b and Socs3 from the Affymetrix^® ^GeneChip^® ^HGU133 A array. In case of multiple probe sets for the same gene, as was the case for Cd40, we chose the most similar in gene sequence mapping between the human and mouse genomes. Intensity data was used to compute per-sample Z-scores. The human dataset resulted from three probe sets and 27 samples (1 stimulus, 9 time series) all labeled as "inflammatory".

### Feature Selection

KEP performs the feature selection task through the ADTree algorithm [45] applied to the *Pre-processed Learning Data Set*. The Weka software environment, Ver. 3.5.6 [29], was used with 10-fold cross validation to obtain the *Selected Features Learning Data Set*.

### Model Learning and Performance Estimation

Model Learning and Performance Estimation, applied to the *Selected Features Learning Data Set*, is concerned with the training and estimation of the classification performance of the following DM models; *ZeroR*, *Nearest Neighbour*, *C4.5*, *Logistic*, *Multi Layer Perceptron*, *Naïve Bayes*, *Random Forest*, *Support Vector Machines *and *Tree Augmented Naïve Bayes*. *ZeroR *uses the majority criteria to classify a sample, i.e. it classifies each sample according to the majority of the class distribution. It is useful to provide a baseline measure of performance. *Nearest Neighbour *[46] (*IB*-k with k = 3 and default learning parameter values) is a *k *nearest neighbour algorithm. *C4.5 *[47] (*J48 *with default learning parameter values) is a decision tree, *Logistic *[48] is a multinomial logistic regression model with a ridge estimator (default learning parameter values), *Multi Layer Perceptron *[49] (*MLP *with default learning parameter values) is a feed-forward neural network. *Naïve Bayes *[49] (*NB *default learning parameter values) is a widely used supervised classifier. *Random Forest *[50] (*RF *with default learning parameter values) is the well-known supervised classification model from Leo Breiman. *Support Vector Machines *[51] (*SOM *with the *puk *kernel and default learning parameter values) are widely used in Bioinformatics and *Tree Augmented Naïve bayes *[52] (*TAN *with default learning parameter values) is a parsimonious version of Bayesian Networks. To evaluate and compare the quality of the DM models, the following performance measures were determined: *Precision*; *Recall*; *F-measure*; *ROC; *and *Accuracy*. To reduce the risk of overfitting, the *n-fold cross validation *schema was repeated *s *times. In brief, each replicate is associated with a different value of the seed responsible for the random partitioning of the *Selected Features Learning Data Set*. The mean values, across ten replicates (*s *= 10), of the performance measures estimated through the 10-fold cross validation (*n *= 10), are summarized in the *Learning Performance Report*. The minimum (*min*), mean (*mid*) and maximum (*max*) values of the considered performance measures are computed.

### Validation

DM models were validated, through the Validation task, by exploiting the *Selected Features Validation Data Set*. This data set was obtained by applying the same filters as applied to the *Learning Data Set *to the *Validation Data Set*, and by using only those features which were selected through the Feature Selection task applied to the *Pre-processed Learning Data Set*.

### Functional Gene Selection

Functional Gene Selection (network analysis) determines the biological significance of the selected gene expression signature. This task was performed using the Ingenuity Pathway Analysis (IPA) software package Ver. 8.0 and content Ver. 2802 which returns graphical representations of the molecular relationships between input molecules. The IPA GUI is exploited to perform the following actions; **i) **to search the corresponding object in the manually curated Ingenuity's Knowledge Base (IKB) in which the gene symbol is associated with probe set identifiers (Table 2), **ii) **to use the selected genes as input into the IPA Core Analysis; **iii) **to find direct and indirect relationships between the genes (network parameters: *a) *number of molecules per net equal to 35; *b) *25 nets per analysis) through the analysis algorithm; **iv) **to edit the retrieved network (*e.g*. to delete peripheral nodes) and, to provide a statistical report concerning relevant pathway nets together with their functional analysis.

The significance of the association between the list of genes and the canonical pathway retrieved by IPA was assessed in two ways: **i) **the ratio between the number of molecules from the list that map to the pathway and the total number of molecules that map to the canonical pathway. **ii) **Fisher's statistic was used to compute the probability value of the null hypothesis, i.e. the probability that the association between the genes included in the list and the canonical pathway is explained by chance alone. The goal of this task is twofold: first to find an explanation for the genes which were selected by the Feature Selection task and which are included in the IPA output, and second to understand the reason why some genes, which were selected by the Feature Selection task, are not included in the IPA output. Then, the *Reduced set of Features*, consisting of the genes included in the IPA output and/or which are believed to be wrongly not included in the list of the selected genes is formed. The *Reduced set of Features *is then used to perform a new validation of DM models.

#### Microarray accession numbers

All microarray data are available from the ArrayExpress database http://www.ebi.ac.uk/arrayexpress/ under the following accession codes. Microarrays data accession number on MGU74Av2 arrays is E-MEXP-2715. Microarray on MOE430A and MOE430A 2.0 have the following accession numbers for reviewer:

*Bordetella pertussis *(ID: Reviewer_E-MEXP-3160 PW: fsEa22df), *Bordetella parapertussis *(ID: ID:Reviewer_E-MEXP-3156 PW: o5TTIG2b), *Listeria monocytogenes *(ID: Reviewer_E-MEXP-3159 PW: habdgpze), *Listeria innocua *(ID: Reviewer_E-MEXP-3158 PW: ojiep0qb), *Lactobacillus paracasei *(ID: Reviewer_E-MEXP-3157 PW: mmcbtpma), *Lactococcus lactis *(ID:Reviewer_E-MEXP-3162 PW: 3vvnihhg)

## Authors' contributions

GTF participated in data collection, pre-processing and participated in writing the manuscript. VV analyzed the data. PRC participated in generating the microarray data. FZ participated in the design of the study, data pre-processing and writing the manuscript. FS designed the KEP protocol, and participated in writing the manuscript. MF participated in designing the study, coordinating the study, generating microarray data and writing of the manuscript. All authors have read and approved the final version of the manuscript.

## Supplementary Material

Additional file 1 **Detailed list of the experiments and array types**. Click here for file
